# Dose reduction in digital radiography based on the significance of marginal contrast detectability

**DOI:** 10.1002/acm2.13230

**Published:** 2021-03-26

**Authors:** Alexander W. Scott, Yifang Zhou, Di Zhang, Nader Binesh, Christina Lee, Mark Bosteder

**Affiliations:** ^1^ Department of Imaging Cedars‐Sinai Medical Center Los Angeles CA USA

**Keywords:** digital radiography, radiation dose, exposure index

## Abstract

The performance of three digital detectors was measured at two exposure index (EI) levels in terms of the effect on features at the borderline of detectability. The null hypothesis was that there would be no statistically significant difference in the CNR of marginally visible features of a baseline‐ (2.2 µGy) and reduced dose (1.4 µGy) images. The experiment used three digital detectors and a phantom composed of an aluminum contrast‐recovery plate, with features of varying diameters and hole depths, which was placed between the detector/grid and 5–20 cm Lucite. Exposures were made using a kVp between 55 and 110 corresponding to the Lucite thickness and a mAs producing an EI of approximately 220 or 140. Images were acquired for all detectors, EI values, and all Lucite thicknesses, then scored by a team of physicists and technologists in terms of feature visibility for each feature size. Contrast‐to‐noise ratio (CNR) was calculated for each feature using an ROI over the feature and a local background annulus. The uncertainty in the CNR was determined by sampling the background at each feature size, finding residuals from an overall background fit, and then calculating a standard deviation in the noise for each size. The marginal feature pair for each feature size bracketed the reader score. The difference between the CNR values of corresponding marginal features in EI‐paired images was significant (*P* < 0.05) for one detector and not significant (*P* > 0.05) for marginal features of the other two. Based on both reader scoring and CNR measurements of phantoms, patient doses can be lowered by 30% for those two detectors without a statistically significant difference in lesion perceptibility of the marginally visible feature, while for the other detector there was a statistically significant change in marginal feature detectability and dose reduction was not recommended.

## 1 INTRODUCTION

The transition in imaging media from screen‐film to digital detectors decoupled dose and image quality. Screen‐film exhibits a loss of contrast when the exposure lies on the shoulder and toe regions of the Hurter‐Driffield (H‐D) curve, meaning that acceptable image quality corresponds to a limited range of doses to the film.^1^ In comparison, digital detectors have a wide dynamic range and it is no longer obvious how much radiation exposure is needed to produce a diagnostically acceptable image. Although this has historically raised concerns of “dose creep,”^2^ digital detectors have led to dose reduction while maintaining acceptable image quality.^3^ Following the principle of minimizing dose (As Low As Reasonably Achievable — the ALARA principle)^4^ for radiography, it is important to define an image quality metric so that the minimum dose meeting the metric can be used.

Although clinical diagnostic quality of an image can be subjective, the physical characterization can be described in terms of noise and contrast between features. The relevant figure of merit for optimizing image quality is therefore the contrast‐to‐noise ratio (CNR), also called the signal‐difference‐to‐noise‐ratio (SdNR).^5^ One prominent strategy for optimizing the image quality with respect to dose levels is to use a figure of merit (FOM) of CNR^2^/E, where E is the exposure to the detector. This FOM will optimize the detector energy response, such as finding the optimal kVp per Lucite thickness, since the tube current (mAs) has been canceled out. It also somewhat balances the image quality improvement with a penalty for increasing patient dose; however, it does not optimize the dose itself for a diagnostic imaging task.

The determination of marginal feature visibility across a range of features has been studied using a contrast‐detail curve, often employing the CDRAD phantom (Artinis Medical Systems, Elst, the Netherlands).^6^, ^7^ This phantom has an array of cylindrical holes with different depths and diameters, as described in its user manual.^8^ The image quality FOM used with this phantom is a software calculation that is inversely related to the sum of the products of the marginal features’ diameters and depths.^9^ This FOM increases as the features identified as marginal by the software become more subtle. However, the clinical significance of this FOM is not clear. It is dose dependent, so decreasing the dose will reduce the FOM, but there is little guidance on how much change in the FOM can be tolerated clinically; the Institute of Physics and Engineering in Medicine (IPEM) recommended a maximum 30% deviation from baseline.^10^ One recent paper^11^ looked at the significance of change to this FOM using the standard deviation of the results and found that changes were unlikely to be reliably detected at less than a 50% dose change. The question remained unanswered, how meaningful is the reduction in image quality when the dose is lowered by a certain amount?

Consider the situation where, instead of optimizing the FOM of CNR^2^/E, the lowering of patient dose is emphasized while requiring acceptable diagnostic quality. The choice of features to represent acceptable diagnostic quality is important, as some hypothetical features will have high enough CNR that they will be visible over the clinically relevant range of detector exposures, and some features will have low enough CNR that they will never be visible over a clinically relevant exposure range. It is reasonable, therefore, to establish a clinical baseline dose and resultant image quality where the features at the borderline of visibility (henceforth called “marginal features”) are defined and further dose optimization should be compared to this baseline. This scenario may be more clinically relevant, where patient radiation safety is touted but image quality only needs to meet a minimum threshold that is task dependent. In this situation, there is a feature‐specific baseline CNR (CNR_B_) and reference patient dose resulting in detector exposure (E_B_) producing acceptable image quality. If the patient dose is lowered so that E < E_B_ and CNR < CNR_B_, will the ability to make a clinical diagnosis based on low‐contrast features be lost?

One approach to addressing the significance of the change in image quality between baseline and reduced dose is to utilize a statistical method proposed in computed tomography (CT) for determining low‐contrast detectability.^12^, ^13^ This method uses background sampling with a large number of matrix elements the size of the feature of interest to obtain the mean pixel value from each element at that spatial scale. If the mean values follow a normal distribution, a quantitative measure of the visibility of a low‐contrast feature over background can be defined, using the criterion that the feature contrast should be 3.29 times the standard deviation of the background values (representing a 90% confidence limit, with 5% probability tails beyond this). Conceptually, this can be applied to the visibility of a change in feature contrast relative to the background as well.

The goal of this research was to determine if there was a statistically significant difference in the low‐contrast detectability of reader‐determined marginal features at very different exposure indices. The null hypothesis was that lowering the EI by 30% from the institutional baseline would produce no statistically significant difference in the CNR of the marginal features of an image quality phantom.

## 2 MATERIALS AND METHODS

The overarching goal was to determine if a change in CNR for marginal features of a contrast‐detail phantom was statistically significant when the dose was lowered by 30% from baseline. The analysis of the images, post‐acquisition, required some distinct steps. First, the marginal features were determined by individuals associated with x‐ray imaging who scored the low‐contrast features for visibility. Second, the contrast was determined by drawing regions‐of‐interest (ROIs), at a spatial scale corresponding to the feature size, over and around the feature. The noise was the average pixel noise from sampling over the image. Then, the variance in the background at different spatial scales was determined from the variance in background means from size‐specific ROIs. Finally, a chi‐squared statistical test was applied to see if the difference in CNR for the corresponding marginal features in baseline‐ and low‐dose images was significant compared to the ratio of variance in background means to pixel noise at that spatial scale. Each of these analysis steps is described below.

### 2.1 Image acquisition

**Fig. 1:**
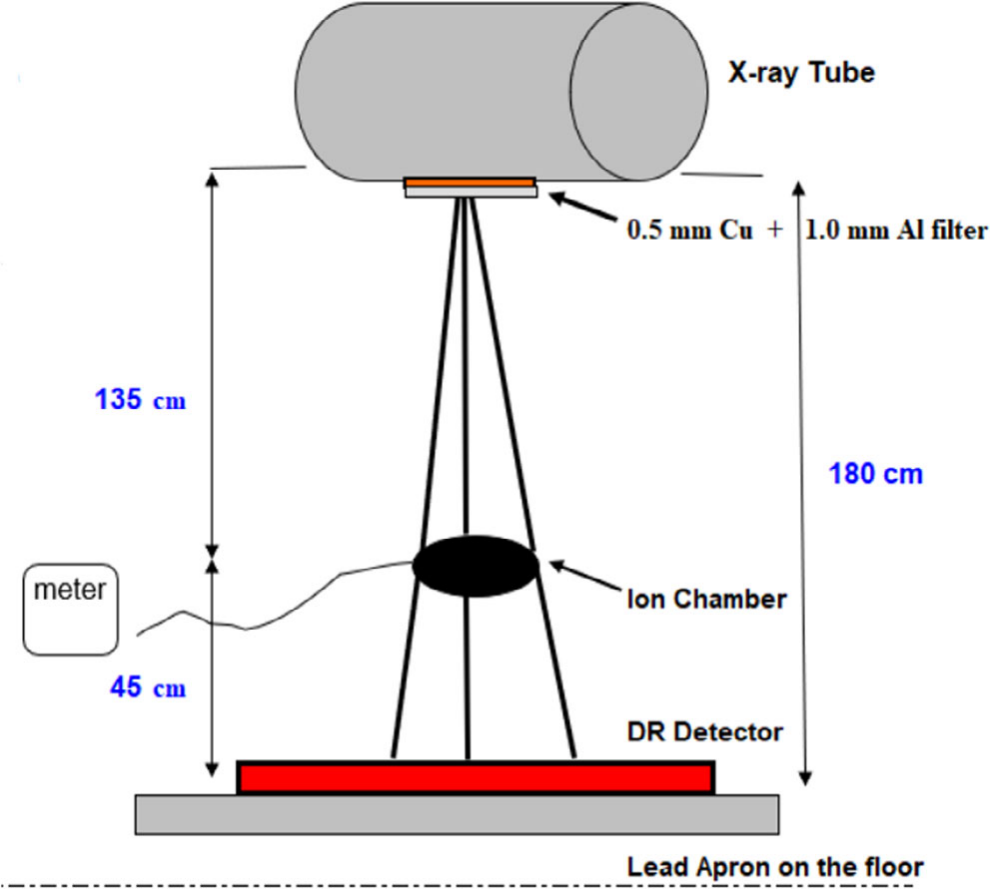
The configuration for testing the exposure index calibration of a digital detector, in accordance with AAPM TG 116.

The experiment was conducted using a Siemens Multix x‐ray room (Siemens Medical Solutions USA, Malvern, PA) retrofitted with both a Carestream DRX‐1c detector and a Carestream DRX‐Plus detector, and also a portable x‐ray unit from a third‐party vendor with a CXDI‐710c digital detector manufactured by Canon. All three were cesium iodide (CsI)‐based digital radiography detectors. Both x‐ray generating devices were acceptance‐tested and had annual physics QC tests performed, and the exposure index calibration for all three detectors was tested at acceptance in accordance with AAPM TG 116 and the manufacturer’s recommendations for creating RQA5 beam conditions (Fig. 1)^2^. Carestream defined their exposure index as $EI\;=\;2000\;+\;1000\cdot \log_{10}\left(X\left\lfloor \mathrm{mR}\right\rfloor \right)$, where *X* is the exposure in units of mR, and specifies beam conditions of 80 kVp with 0.5 mm Cu and 1.0 mm Al in the beam. Canon followed the IEC standard of defining exposure index as $EI\;=\;100\cdot K\left\{\mu Gy\right\}$, where *K* is the air kerma in units of µGy and specifies beam conditions of 70 kVp with 0.5 mm Cu and 1.0 mm Al in the beam. EI calibrations were determined to be accurate at acceptance within 10%. Table 1 shows a comparison of beam conditions and exposure index definitions for calibration checks of the detectors.

**Table 1:** Vendor information on exposure index (calibration beam quality and EI definition) for the three digital detectors.

Detector	Calibration	Exposure Index
DRX‐1c	80 kVp, 0.5 mm Cu + 1.0 mm Al	$EI=2000+1000\cdot \log_{10}\left(X\left\lfloor \mathrm{mR}\right\rfloor \right),$
DRX‐Plus	80 kVp, 0.5 mm Cu + 1.0 mm Al	$EI=2000+1000\cdot \log_{10}\left(X\left\lfloor \mathrm{mR}\right\rfloor \right),$
CXDI 710C	70 kVp, 0.5 mm Cu + 1.0 mm Al	$EI=100\cdot K\left\{\mu Gy\right\},$

**Fig. 2:**
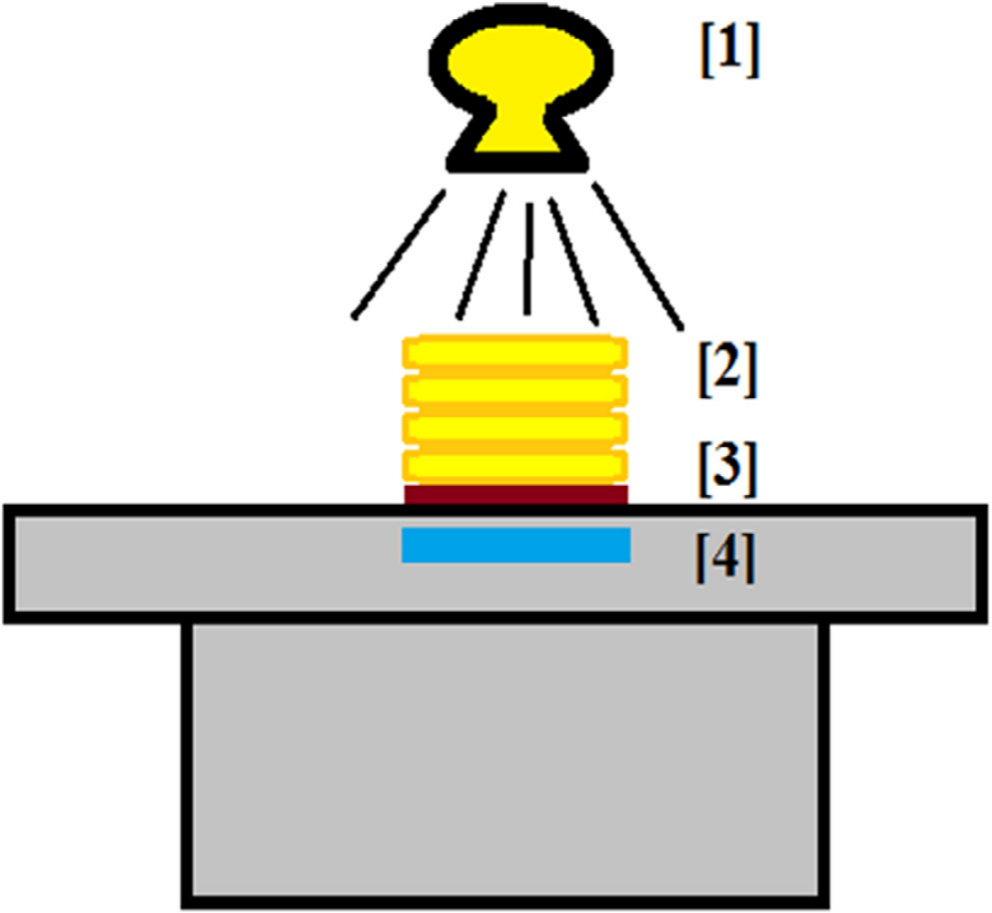
The configuration for testing the image quality at high and low exposure index. Element [1] is the x‐ray imaging device (either Siemens Multix room unit or portable Canon unit), element [2] is the Lucite thickness (varying from 5 cm to 20 cm), element [3] is the aluminum contrast‐detail test object, and element [4] is the detector and grid (either a Carestream DRX‐1c / DRX‐Plus with a Siemens grid or a Canon CXDI 710C and portable grid).

Image quality phantoms were constructed using variable thicknesses of Lucite and a Gammex (Sun Nuclear Corp, Melbourne FL) model 1151 aluminum contrast‐detail recovery plate with milled low‐contrast targets in a matrix of different diameters and hole depths (see Table 2); a diagram of the experimental setup is shown in Fig. 2. The Lucite stack was 30 cm × 30 cm in area with variable thicknesses of 5 cm, 7.5 cm, 12.5 cm, or 20 cm. The image quality phantom was placed either on the table with the detector in the bucky (Siemens Multix room) or directly on the detector/grid (Canon portable), with the image quality object in contact and the Lucite distal to the detector, so that features were in a constant position as the Lucite thickness varied. Properties of the grids used are listed in Table 3. The source‐to‐detector distance used was 100 cm. The phantom was centered in the x‐ray beam and the beam collimated to just inside the area covered by the Lucite. The beam quality was 3.06 mm Al at 80 kVp for the Canon device and 3.28 mm Al at 80 kVp for the Siemens device.

**Table 2:** Gammex model 1151 aluminum contrast‐detail recovery phantom has a matrix of holes, arrayed 10x10. The phantom was imaged so that depth (contrast) varied by rows and diameter varied by columns. Hole depth and diameter are given in mm.

Feature designation	1	2	3	4	5	6	7	8	9	10
depths (mm)	2.29	1.63	1.14	0.76	0.51	0.43	0.36	0.28	0.20	0.13
diameters (mm)	7.92	5.56	4.75	3.96	3.18	2.39	1.60	1.19	0.84	0.58

**Table 3:** Grid information for the two experimental designs. The Carestream DRX detectors were mounted in a table bucky, while the Canon CXDI detector was sheathed in a portable grid.

Detector	Grid ratio	Strip density	Focal distance
DRX‐1c	12:1	40 lp/cm	113 cm
DRX‐Plus	12:1	40 lp/cm	113 cm
CXDI 710C	8:1	52 lp/cm	100 – 180 cm

Two images were produced for each phantom build using a manual technique: a kVp between 55 and 110 as appropriate to the Lucite thickness^14^ (see Table 4) and a mAs producing an EI of approximately 220 or 140. The two target EI values were chosen based on the baseline EI and the experimental target EI, but the actual EI values had to be approximate because of the granularity of the mAs stations. Acquisition parameters and the resultant exposure index values are provided in Table 5. There were a total of 24 images produced for the three detectors, the four Lucite thicknesses, and the two EI levels. The images were acquired using a minimal processing algorithm such as “Pattern.” Gain and offset calibrations were applied, but the image was not processed based on assumptions of anatomy.

**Table 4:** Corresponding kVp and Lucite thicknesses when evaluating detector performance, provided by Carestream. The same table was also used to evaluate all detectors.

X‐ray Energy (kVp)	Lucite thickness (cm)
50	5
65	7.5
85	12.5
110	20

**Table 5:** Exposure information for all the detectors and Lucite thicknesses, including kVp, mAs, and the exposure index. The Carestream EI is followed by the IEC‐defined EI in parentheses for easier comparison with the Canon results.

Detector	Low‐dose exposure			High‐dose exposure		
	kVp	mAs	EI	kVp	mAs	EI
DRX‐1c	50	2.5	1226 (147)	50	3.6	1392 (215)
	66	1.25	1201 (139)	66	2.0	1415 (227)
	85	1.25	1183 (133)	85	2.0	1399 (219)
	109	2.2	1219 (145)	109	3.2	1390 (214)
DRX‐Plus	50	3.2	1221 (145)	50	5.2	1417 (228)
	66	1.6	1197 (137)	66	2.5	1399 (219)
	85	1.6	1185 (134)	85	2.5	1405 (222)
	109	2.5	1210 (142)	109	4	1415 (227)
CXDI 710C	55	32	142	55	40	208
	65	12.5	128	65	20	205
	85	5.0	120	85	8.0	207
	110	6.4	146	110	10	235

### 2.2 Reader scoring

**Fig. 3:**
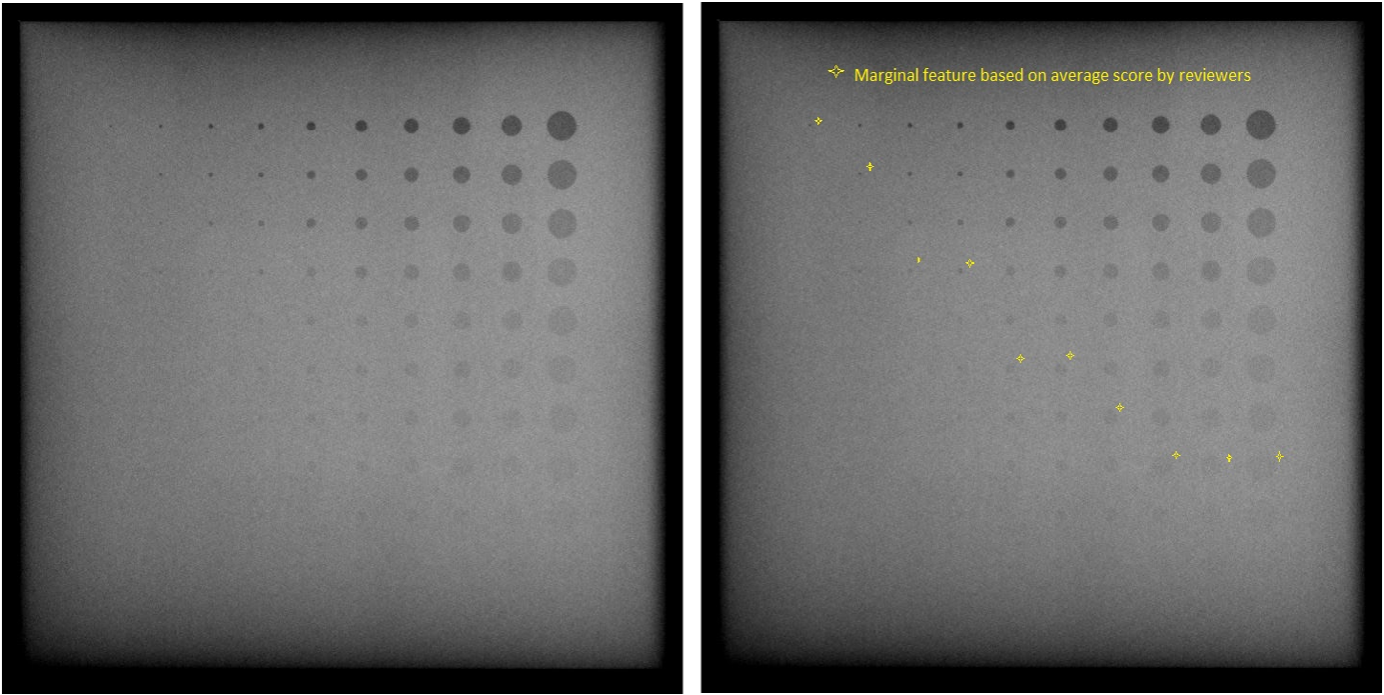
The aluminum contrast‐detail phantom, oriented as scored by readers. Iso‐diameter features are organized by columns from right to left (decreasing size) and iso‐contrast features are organized by rows from top to bottom (decreasing contrast). As an example, feature $f_{1,1}$ would be the top right, largest size, and greatest contrast. The figure on the right is an example of reader scoring with a mark for each column at the last visualized feature; this score is 8‐8‐8‐7‐6‐6‐4‐4‐2‐1, starting from the right and counting down in each column.

In order to identify the marginal features used in the analysis, the images were scored for the last resolvable feature of each feature size, which was the lowest contrast feature for each diameter (column) that appeared circular. The low‐contrast features were defined as $f_{i,j}$, where $i\in \left\{1,2,\ldots ,10\right\}$ beginning with the largest diameter and $j\in \left\{1,2,\ldots ,10\right\}$ beginning with the highest contrast. A representative image of the contrast‐detail phantom and a reader score of the image are shown in Fig. 3. The images were ordered pseudo‐randomly using the Excel function RAND() and printed from DICOM to pdf so that the images could be viewed at personal workstations consistently by all the readers in terms of size, contrast, and resolution. The images were scored by a group of physicists and technologists; the Carestream images were scored by four physicists (experience ranging from 4 to 10+ years) and two technologists (experience ranging from 5 to 10+ years), while the Canon images were scored by four physicists (experience ranging from 9 to 10+ years) and four technologists (experience ranging from 5 to 10 + years). The difference in scoring team composition between Canon and Carestream is due to the fact that images were acquired at separate times and there was personnel turnover in between. The image scores were reported in the form of $f_{1,j}‐f_{2,j}‐\ldots ‐f_{10,j}$, where *j* represents the lowest contrast feature that was distinguishable. A feature score mean (average j) and standard deviation for each i were calculated from the collected reader data. The marginal features for each size were then determined so as to bracket the mean reader score; for example, if the mean reader score for a group of 10 features in column i were 7.1, then the marginal features for that column i would be $f_{i,7}$ and $f_{i,8}$. To serve as an additional check of the quantitative results, the differences in scores for the baseline‐ and low‐EI image pairs for each reader were calculated and the standard deviation among readers for the score difference was found.

### 2.3 Feature contrast measurement

**Fig. 4:**
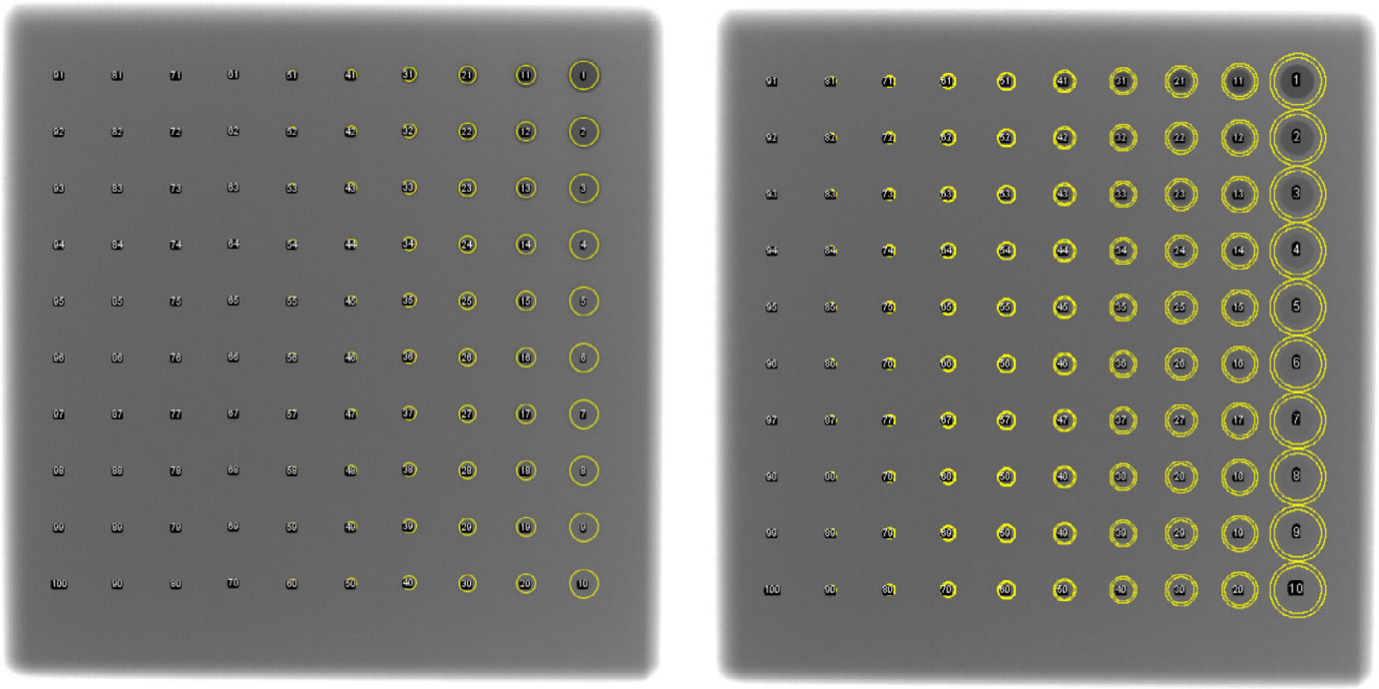
A representative image of the contrast‐detail phantom with feature ROIs (left) and annular rings for local background (right). The ROI sizes are reduced to correspond to the feature sizes.

In addition to reader scoring, the images were quantitatively evaluated to measure the CNR of each feature using the ImageJ software (National Institutes of Health, Bethesda, MD). To determine the contrast for each $f_{i,j}$, the pixel intensity $S_{i,j}$ was measured using an ROI drawn over the feature using an ImageJ macro, designed so that the ROI would be of slightly smaller size than the target area as reported by the phantom specifications and positioned to just fit inside the feature. The local background $B_{i,j}$ was measured with an annular ring surrounding the feature $f_{i,j}$ and of similar size to the feature ROI. For illustration purposes, a representative image of the feature ROIs and local background ROIs is shown in Fig. 4. However, the background pixel intensity varied with a gradient across the image and the gradient was sufficiently steep in places to prevent the use of the annular ring pixel noise $N_{i,j}$ as the noise when calculating the CNR. It was ultimately decided to use a global but size‐dependent value of the noise for the CNR of each feature in the image, as described in the next subsection.

### 2.4 Background variance determination

**Fig. 5:**
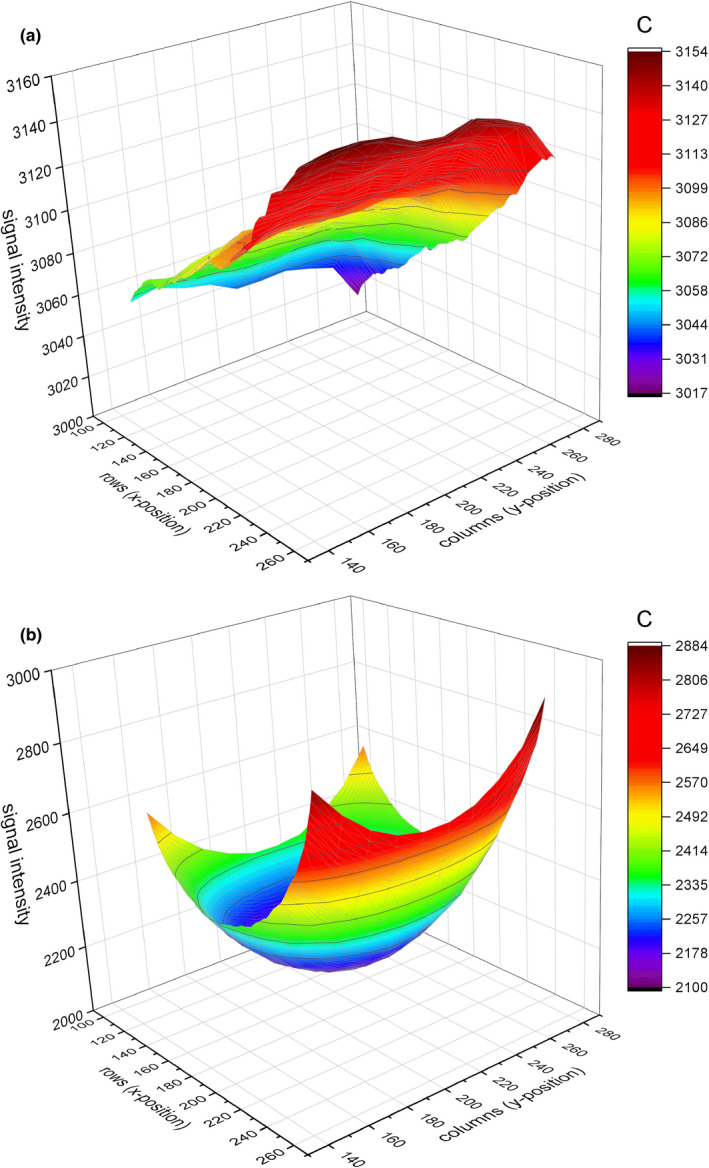
(a and b) representative images of the background pixel value distribution. Shown are the DRX‐Plus detector, 8”, low‐EI image (left) and CXDI‐710c detector, 8”, low‐EI image (right).

The process for determining the spatial scale‐specific background variance utilized the distribution of the means of an ensemble of size‐specific background samples, which was fitted to find the standard deviation of that distribution^12^, ^13^. This standard deviation served as the statistical uncertainty in the contrast at that spatial size. However, it was noted that there was a DC component of the background which consisted of a signal gradient across the images (see Fig. 5a,b); this gradient was due to a combination of the heel effect and the scatter distribution near the edge of the Lucite stack and introduced a position‐dependent element to the background means which artificially widened the normal distribution. To remove this position dependence, the gradient component was subtracted from all pixel values before evaluating the background variance. The gradient for the Carestream detector images turned out to be too complex for a contour fit across the entire image because of variability in both x and y directions, so the background was fitted for specific rows where the background sampling was done. The contour fit for the Canon detector images was successful, but the same row‐by‐row fitting was used for consistency with the Carestream images. After subtracting the fit, the background samples had a variance independent of image coordinates.

**Fig. 6:**
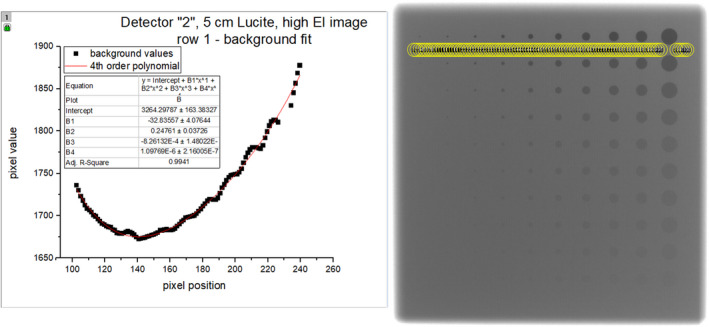
A representative image of the contrast‐detail phantom with oversampled ROIs for the background fit beneath row 1 (right) and the distribution of mean ROI values with fourth‐order polynomial fit (left). The ROI size for the background fit is the size of the largest feature ROI. The sampling and fit were performed for all the 10 rows.

First, a polynomial fit (either 4^th^ or 5^th^ power depending on whether the background distribution appeared even or odd) was determined in one‐dimension in the background space below each row of iso‐contrast elements (between $f_{i,1}$ and $f_{i,2}$, between $f_{i,2}$ and $f_{i,3}$, and so forth) using oversampled ROIs of the same area as the ROI for the largest feature $f_{1,j}$. The polynomial fit was not performed at multiple sizes since the shape of the DC component was not dependent on the sampling size. For illustration purposes, a representative image of one row of oversampled ROIs on the contrast‐detail phantom image and the resultant background distribution and polynomial fit for that region are shown in Fig. 6.

Next, the background was sampled at 100 locations with an ROI of the same area as the ROI for the largest feature size i=1, with the sampling evenly distributed along the coordinates covered by the polynomial fit; the process was repeated for the nine other feature sizes i=2…10 to yield 100 background samples for each of the feature sizes. The pixel noise from all background sample ROIs of the same size was averaged for use as the global noise in the CNR calculations for that feature size i, meaning the value of the noise $N_{p,i}$ was size specific but independent of image coordinates.

**Fig. 7:**
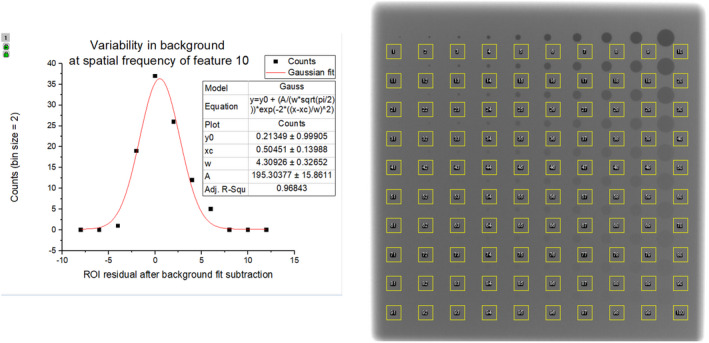
A representative image of the contrast‐detail phantom with background ROIs for the residuals fit along all rows (right) and the distribution of background fit‐subtracted mean ROI values (background residuals) with a Gaussian fit (left). The origin function used for Gaussian fits has a parameter “w” which is 2σ. The value of σ parameterizes the variability in the background at that ROI size. The sampling and fit were performed for all the 10 feature sizes.

Additionally, the background sample means for all the 10 sizes were reduced by the result of the polynomial fit at the corresponding ROI coordinates to remove the DC component of the background. After subtraction, the background sample means were residuals to the background fit; a histogram of the background residuals was generated for each ROI size $i\in \left\{1,2,\ldots 10\right\}$, which was well‐fitted by a Gaussian in all cases (R^2^ > 0.90). For illustration purposes, a representative image of the background sample ROIs and the resultant residual histogram and Gaussian fit are shown in Fig. 7. The $\sigma_{i}$ from the Gaussian fits of samples of size i was used to determine the uncertainty in the CNR calculation for each $f_{i,j}$, as it described the statistical variance in the background at size i.

### 2.5 Calculating CNR change and uncertainty

The final calculation was to find the CNR and CNR difference ($\Delta CNR_{i,j}$) for corresponding marginal features in the EI image pair (images from same detector and phantom but at baseline and lower dose). The $CNR_{i,j}$ was calculated as the difference in feature ROI and annular ring ROI values divided by the size‐specific pixel noise: $CNR_{i,j}=\left(S_{i,j}‐B_{i,j}\right)/N_{p,i}$, while the uncertainty in $CNR_{i,j}$ was the standard deviation from the Gaussian fit of background means divided by the size‐specific pixel noise: $\sigma CNR_{i.j}=\sigma_{i}/N_{p,i}$. The uncertainty in $\Delta CNR_{i,j}$ was the sum in quadrature of the individual uncertainties from the EI image pair. The significance of the finding was determined by using the chi‐squared statistic, with a p‐value of 0.05, for the $\Delta CNR_{i,j}$ of the marginal features on the contrast‐detail phantom images. The significance was determined for all marginal features in the image $i\in \left\{1,2,\ldots 10\right\}$ and separately for the five largest features $i\in \left\{1,2,\ldots 5\right\}$.

## 3 RESULTS

**Fig. 8:**
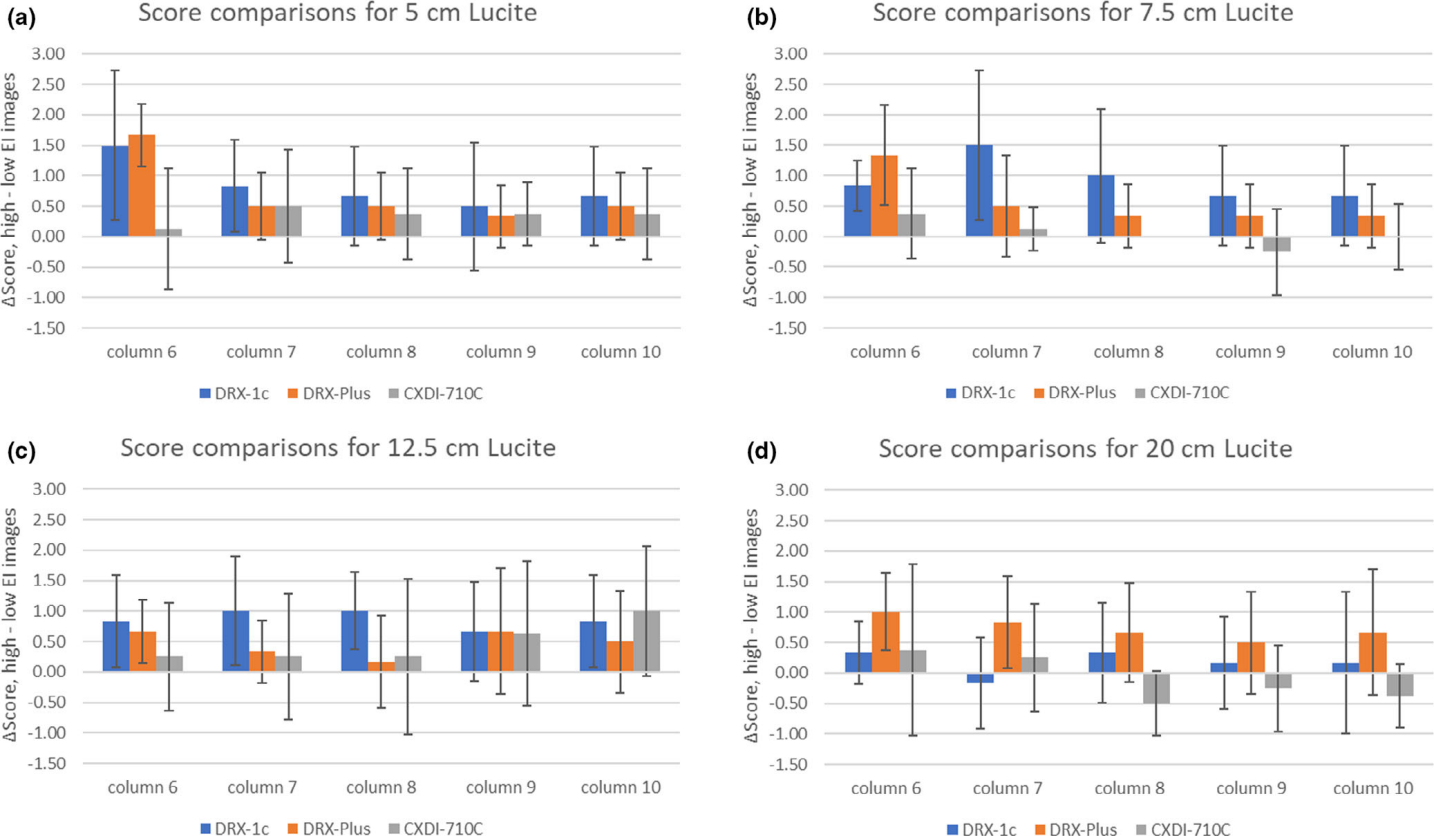
(a–d) These four figures for the four Lucite thicknesses show the median reviewer score difference between high‐ and low‐EI images, for the largest size features (columns 6‐10) of the aluminum contrast‐detail recovery phantom. The standard deviation is based on the score difference for that feature.

The difference in reader scores for EI image pairs was compared across the three detectors with results displayed in Figs. 8(a)‐8(d). The scoring difference between EI image pairs for the five largest diameter features was less than or comparable to the standard deviation of the scoring difference. The exception was for the DRX‐Plus detector, 5 cm phantom, feature size 5, where the scoring difference was three times the standard deviation. Of 60 mean scoring differences among the three detectors, only two features (including the aforementioned one) had a ratio relative to the standard deviation greater than 2.0 and no single reader was responsible for the score difference.

To evaluate the readers relative to each other, an analysis of variance (ANOVA) single factor statistical test was performed to determine if the readers’ scores were consistent at the 5% level. For all the three detectors, the individual readers were not consistent with each other at the 5% level, but the mean tech group score was consistent with the overall mean and the mean physicist group score was also consistent with the overall mean.

**Fig. 9:**
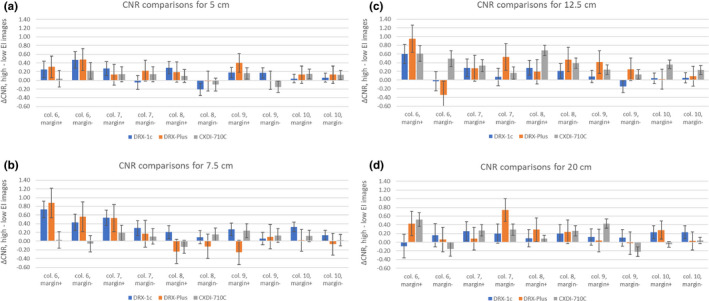
(a‐d) These four figures for the four Lucite thicknesses show the CNR difference between high‐ and low‐dose images for the marginal features of the contrast‐detail phantom images. The CNR differences of the marginal features are labeled “margin+” and “margin‐” depending on whether the feature has higher or lower contrast than the mean visible feature. The error bars come from the distribution of background values, which affects the precision of the CNR.

The difference in the CNR between corresponding features of EI‐paired images was found for the marginal features of each feature size. Figs. 9(a)‐9(d) show the difference in CNR between high and low EI for the five largest features. The p‐value and reduced chi‐squared are included in Table 6 for each detector and phantom combination; only the data from the five largest feature sizes were used to calculate the p‐value and chi‐squared.

**Table 6:** For each detector and Lucite thickness is shown the chi‐squared and p‐value for the significance of the CNR differences of the five largest marginal feature sizes. A p‐value of 5.0E‐2 or less indicates a significant result, meaning that there is a statistically significant difference in CNR between the high‐ and low‐EI image pair.

		DRX‐1c	DRX‐Plus	CXDI‐710C
5 cm Lucite	reduced χ^2^	2.47	1.29	1.07
	*P* ‐value	8.27E‐03	2.37E‐01	3.84E‐01
7.5 cm Lucite	reduced χ^2^	5.51	1.61	0.84
	*P* ‐value	9.00E‐05	1.06E‐01	5.75E‐01
12.5 cm Lucite	reduced χ^2^	1.67	2.03	10.15
	*P* ‐value	9.09E‐02	3.18E‐02	1.00E‐05
20 cm Lucite	reduced χ^2^	0.73	1.57	5.18
	*P* ‐value	6.79E‐01	1.19E‐01	1.00E‐05

For the DRX 1‐C detector, there was a significant change in CNR for the two smaller thicknesses of Lucite when lowering the dose, but no significant drop in CNR for the two larger thicknesses of Lucite. For the DRX‐Plus detector, there was no significant drop in CNR for 5 cm, 7.5 cm, and 20 cm thicknesses of Lucite when lowering the dose; the drop in CNR was significant for 12.5 cm Lucite. For the CXDI‐710c, there was no significant drop in CNR for the two smaller thicknesses of Lucite when lowering the dose and a significant drop in CNR for the two larger thicknesses of Lucite.

## 4 DISCUSSION

As part of this research a new generation detector was evaluated, which was reported to have a 60% reduction in dark noise and 25% increase in sensitivity, with a corresponding improvement in detective quantum efficiency (DQE) at high spatial frequencies (3 mm^‐1^) and low doses (0.97 µGy).^15^ To complement this evaluation, two other detectors were compared regarding the visibility of marginal features when lowering the detector exposure from baseline. The institution’s historical baseline exposure index (EI) of 220, corresponding to a detector air kerma of 2.2 μGy, was established by a collaboration of technologists, radiologists, and medical physicists. The clinical practice desired to set the target EI for the new generation detector to 140, which would result in a 30% reduction of radiation dose to patients.

It was decided to focus on the five largest features instead of all 10 feature sizes. The reason for separating out the largest features was that the very smallest features were only one or two pixels in diameter and the confidence in the measurement matrix aligning with the feature arrangement within two pixels was considered to be low. In addition, there was no clinical need to identify lesions of this size.

The reader scores overwhelmingly showed a smaller change in score between EI image pairs than the standard deviation of reader scores. That suggested the significance of the change in image quality for the marginal features was likely to be low. To quantitatively assess the image quality changes when dropping the dose from baseline, a chi‐squared statistic was used to find the significance in the change in CNR compared to the uncertainty. The chi‐squared number itself was the ratio of CNR to $\sigma /N_{P}$; the pixel noise canceled out, resulting in the change of contrast divided by the size‐specific background noise. To result in significant change (p >.05), the mean ratio of contrast change to background noise needed to exceed approximately 1.4 for 10 marginal features from sizes $i\in \left\{1,2,\ldots 5\right\}$. This is very similar to using the background noise to determine the visibility of a feature with contrast of $\Delta S_{i,j}‐\Delta B_{i,j}$, pixel noise of $\sigma_{i}$, and a threshold for detectability of 1.4.

For the DRX 1‐C detector, the loss of CNR was significant for the two smallest Lucite thicknesses. The uncertainty in the CNR for these two phantoms started at 0.06 for the largest feature size and increased to 0.13 for the smallest. For the two largest phantoms, the CNR uncertainty started at 0.07 and increased to 0.20. The mean CNR difference between like features of EI paired images was comparable for phantoms 5 cm, 12.5 cm, and 20 cm (varying in value from 0.14 to 0.15) so the driver for the lower chi‐squared value for the 12.5 cm and 20 cm phantoms was the greater background variation in the image (higher uncertainty in the CNR). The 7.5 cm phantom was an outlier with greater separation in the CNR of like features in EI paired images (mean CNR difference of 0.31 per feature).

For the DRX‐Plus, the loss of CNR was only significant for the 12.5 cm Lucite phantom. The CNR uncertainty averaged 0.16 for the largest feature and 0.21 for the smallest feature. The background variation was higher for DRX‐Plus than for DRX 1‐C, although the CNR for the same feature was also higher for the DRX‐Plus. The mean CNR difference between like features of EI paired images varied from 0.2 to 0.3, so the driver for the lowered chi‐squared overall was the greater background variation in the image (higher uncertainty in the CNR). Only one of the four phantom builds showed a significant loss of CNR when lowering dose, and the significance of the change in EI for 12.5 cm Lucite almost entirely came from a single point where the CNR difference was 0.95. The CNR was abnormally high for that single feature and was inconsistent with the progression of contrast for the nearby features. Without that unusual feature, the change in EI was not significant as evidenced by the reduced chi‐squared dropping from 2.0 to 1.1. The dependence on one unusual feature suggests this detector would not have experienced any significant loss of CNR with lowered dose if multiple repetitions had been acquired to average out.

For the CXDI‐710c, the loss of CNR was significant for the 12.5 cm and 20 cm phantom, but not for the 5 cm and 7.5 cm phantoms. For the two smaller size phantoms, the CNR uncertainty averaged 0.08 for the largest feature size and increased to 0.13 for the smallest feature size. The mean EI difference was 0.13 for the two phantoms. For the two larger phantoms, the mean EI difference more than doubled to 0.36 and 0.23 (respectively), while at the same time the uncertainty in the CNR dropped somewhat, ranging from 0.05 to 0.14. With a greater difference in feature CNR between EI paired images and lower uncertainty, the p‐values were driven close to zero. This is consistent with anecdotal reports of lower image quality in comparison to other detectors for large patients.

After reviewing the data, some of the difficulties of the analysis would have been lessened if there had been an ensemble of images for each phantom build, technical factors, and detector. One advantage would have been the increased statistical power of multiple images contributing to the statistical uncertainty. Another advantage would have been an improved background subtraction, since the background shape could have been derived from the average over multiple images and the difference between images would be the noise only. Finally, the derivation of the CNR uncertainty using the distribution of size‐specific background ROI means would have been much easier using a subtracted image, where that all that remains is noise. This method would produce a wider Gaussian (by a factor of $\sqrt{2}$) but would produce a cleaner result than using a background subtraction. Unfortunately, the original imaging equipment has been, or is being, retired since the data collection occurred, and some of the readers have moved on from the institution. It would require a future project to collect additional samples for analysis and comparison.

## 5 CONCLUSION

These three detectors, DRX‐1C, DRX‐Plus, and CXDI‐710c, have been used together at the institution for over a year. The technologists used a reduced target EI of 140 for the DRX‐Plus and the image quality has anecdotally still been preferred over the DRX1‐C model and CXDI‐710c at the baseline target EI of 220. Although the three detectors could not be compared directly to each other, their relative low‐contrast performance at a lowered target EI was evaluated with a focus on the marginal features (defined as bracketing the mean reader score for last visualized feature). The DRX‐Plus detector, for three of the four phantoms, showed no significant change in the CNR of the marginal features as the EI was reduced by 0.8 µGy, and also for the fourth phantom when a single anomalous result was removed. The DRX1‐C model, for the two smallest phantoms, showed a significant loss of CNR for the marginal features when reducing the EI. For the larger two phantoms, the CNR loss was not significant because the background variation increased to exceed the signal difference, indicating not that the detector is robust against reduced dose but that the image is too noisy for the loss of signal to matter.

The Canon CXDI results were a little more complicated to interpret. For the two smallest phantoms, the mean CNR difference in like features of EI paired images was comparable to the mean uncertainty in the difference. For the two largest phantoms, the mean CNR difference increased by a factor of 2x to 3x, while the uncertainty in the difference dropped slightly. This was interpreted as the detector performance genuinely worsening for the marginal features as they were starved for signal.

Based on these results, it was recommended to the institution that units with detector DRX‐Plus can drop the detector dose to EI 140 for all cases, that units with the CXDI‐710c detector can drop the detector dose to EI 140 only for thin patients or thin anatomy (reflected in this experiment as thicknesses <10 cm), and that units with detector DRX1‐C should remain at the baseline dose of EI 220. Although this may seem complicated, for a large institution we have technique charts tailored to the individual imaging device and techs assigned to a particular room, so that equipment‐specific requirements have been adopted easily by the majority users.

## AUTHOR CONTRIBUTIONS

Alexander W. Scott involved in experimental design, data acquisition, data analysis, and manuscript drafting. Yifang Zhou, Di Zhang, and Christina Lee involved in data analysis, image scoring, manuscript critique, and approval. Nader Binesh involved in image scoring, manuscript critique, and approval. Mark Bosteder involved in experimental design, image scoring, manuscript critique, and approval.

## CONFLICT OF INTEREST

No conflict of interest.
